# Myocardial Adipose Triglyceride Lipase Overexpression Protects against Burn-Induced Cardiac Lipid Accumulation and Injury

**DOI:** 10.1155/2019/6428924

**Published:** 2019-05-16

**Authors:** Lingfei Li, Xingyue Zhang, Qiong Zhang, Jiezhi Jia, Junhui Zhang, Dongxia Zhang, Huapei Song, Bing Chen, Jiongyu Hu, Yuesheng Huang

**Affiliations:** ^1^Institute of Burn Research, Southwest Hospital, Third Military Medical University (Army Medical University), Chongqing, China; ^2^State Key Laboratory of Trauma, Burns and Combined Injury, Southwest Hospital, Third Military Medical University (Army Medical University), Chongqing, China; ^3^Endocrinology Department, Southwest Hospital, Third Military Medical University (Army Medical University), Chongqing, China

## Abstract

Maladaptive cardiac metabolism is a common trigger of cardiac lipid accumulation and cardiac injury under serious burn challenge. Adipose triglyceride lipase (ATGL) is the key enzyme that catalyzes triglyceride hydrolysis; however, its alteration and impact on cardiac function following serious burn injury are still unknown. Here, we found that the cardiac fatty acid (FA) metabolism increased, accompanied by augmented FA accumulation and ATGL expression, after serious burn injury. We generated heterozygous ATGL knockout and heterozygous cardiac-specific ATGL overexpression thermal burn mice. The results demonstrated that partial loss of ATGL could not relieve burn-induced cardiac lipid accumulation and cardiac injury, possibly due to the suppression of cardiac FA metabolism plus insufficient compensatory glucose utilization. In contrast, cardiac-specific overexpression of ATGL alleviated cardiac lipid accumulation and cardiac injury following burn challenge by switching the substrate preference from FA towards increased glucose utilization. The underlying mechanism was possibly related to increased glucose transporter-1 expression and reduced cardiac lipid accumulation induced by ATGL overexpression. Our data first demonstrated that elevated cardiac ATGL expression after serious burn injury is an adaptive, albeit insufficient, response to compensate for the increase in energy consumption and that further overexpression of ATGL is beneficial for ameliorating cardiac injury, indicating its therapeutic potential.

## 1. Introduction

Severe burn injury leads to substantial hemodynamic and cardiodynamic derangements, which contribute to the development of sepsis, multiple organ failure, and death [1–3]. Myocardial damage following thermal injury is manifested primarily by a decrease in cardiac output with compensatory increments in the heart rate and peripheral vascular resistance, ultimately leading to cardiac dysfunction [4]. The precise mechanisms underlying the development of this cardiomyopathy during serious burn injury are incompletely elucidated. Severe burn injury can trigger a hypermetabolic state that lasts for years following the injury, leading to increased detriment of the patients [5, 6]. There is evidence suggesting that maladaptive cardiac metabolism induced by increased oxygen demand, mismatch between fatty acid oxidation (FAO) and glucose oxidation, and an acquired defect in oxidative phosphorylation are important triggers of cardiac dysfunction under pathological stress [7]. However, the precise changes in cardiac metabolism and the related underlying mechanism need to be further studied.

The heart is unique among organs in the amount of energy required to maintain its mechanical function with or without exogenous stimuli [8, 9]. Under normal conditions, the adult heart is almost exclusively aerobic, with free fatty acid (FFA) as the predominant substrate [10]. During burn challenge, the heart breaks down a large amount of triglyceride (TG) to produce FFA to compensate for the increased energy demand produced by FAO. At the same time, the augmented TG hydrolysis promotes excessive FFA accumulation within the cardiomyocyte [11, 12], which partially inhibits glucose oxidation and may lead to lipid peroxidation of cardiomyocytes through cell death pathways such as necrosis and apoptosis [13–16]. Therefore, the robust metabolic response provides early adaptive protection; however, these remodeling events are deemed maladaptive and may predispose to the ever-rising cardiac dysfunction [17–20]. Thus, a comprehensive understanding of the detailed metabolic processes of the heart after burn injury is required.

Adipose triglyceride lipase (ATGL) is the rate-limiting enzyme that hydrolyzes TG at the first step of fat metabolism [21, 22]. ATGL is predominantly expressed in adipose tissue, skeletal muscle, and cardiac muscle, and its deficiency causes severe cardiac TG accumulation in mice, which leads to a serious condition known as lipotrophic cardiomyopathy, resulting in premature mortality [21, 23, 24]. Consistent with those reports, mutations in the human ATGL gene lead to cardiac steatosis, cardiomyopathy, and heart failure [25, 26]. These findings suggest a possible key role for ATGL in the regulation of cardiac metabolism and dysfunction under multiple pathological stimuli. However, whether ATGL-mediated TG metabolism is involved in cardiac dysfunction after burn challenge needs to be further explored.

To investigate the hypothesis, we first detected the changes in cardiac ATGL expression postburn, and then, heterozygous ATGL knockout (ATGL-Het) and heterozygous cardiac-specific ATGL overexpression (MHC-ATGL Het) mice were used to explore the effect of ATGL on cardiac metabolism and cardiac injury following serious burn injury. We demonstrated, for the first time, that elevated cardiac ATGL expression after serious burn injury was an adaptive, albeit insufficient, response to compensate for the increase in energy consumption and that further overexpression of ATGL helped ameliorate the cardiac injury, thus indicating its therapeutic potential in the prevention and treatment of cardiac lipid accumulation and cardiac dysfunction under pathological stress.

## 2. Materials and Methods

### 2.1. Animal Studies

Homozygous ATGL knockout (ATGL-KO) mice were purchased from the Jackson Laboratory (stock number: 019003, Bar Harbor, ME, USA) and then crossed with wild-type (WT) mice to obtain ATGL-Het mice. Mice with cardiac ATGL overexpression driven by the myosin heavy chain promoter were generated in the laboratory of Rudolf Zechner [23]. Both ATGL-KO and MHC-ATGL Het mice were backcrossed to a C57BL/6J genetic background for more than ten generations. Genotypic identification was performed according to the instructions from the Jackson Laboratory and Rudolf Zechner. Male/female WT littermates were used as controls for experiments with corresponding ATGL-Het or MHC-ATGL Het male/female mice. Mice were housed on a 12-hour light and 12-hour dark cycle with ad libitum access to chow diet and water. Experiments involving animals were performed in accordance with United Kingdom Home Office and European Union guidelines and were approved by the Animal Care Centre and Use Committee of the Third Military Medical University (Army Medical University).

### 2.2. Thermal Burn Experimental Model

After induction of anesthesia with intraperitoneal amobarbital sodium (66 *μ*g/g body weight), the dorsum of each mouse was shaved. Then, the dorsum was placed in a specially constructed plastic mold that exposed approximately 25% of the total body surface area (TBSA) and then immersed in 88°C water for 10 s to produce a full-thickness burn injury and in room temperature water for a sham burn [27]. After inducing the burn injury, each mouse was immediately resuscitated with 1 ml of lactated Ringer's solution given intraperitoneally and allowed water and mouse food ad libitum. The biochemical and metabolic parameters were determined after fasting 8 hours before measurement. The animals were used in experiments 24 hours after the burn challenge.

### 2.3. Histological Analysis

The skin tissue was cut and fixed with 10% formalin, embedded in paraffin, and sectioned at 6 *μ*m thickness. For routine histopathology, sections were stained with hematoxylin-eosin. For apoptosis, sections were stained with the TUNEL (terminal deoxynucleotidyl transferase-mediated dUTP nick-end labeling) kit.

### 2.4. Immunoblot Analysis

The left ventricular myocardium of mice was dissected and homogenized in a tissue protein extraction reagent (T-PER, Thermo Fisher Scientific, Waltham, MA, USA) with protease inhibitor tablets. The lysate was centrifuged at 16,000 g and 4°C for 15 min to remove insoluble protein. SDS-PAGE was carried out after equal BCA-based protein loading for each sample (60 *μ*g) using gradient gels. Separated proteins were transferred to PVDF membranes (Millipore, Darmstadt, Hesse, Germany), blocked with 5% skimmed milk, and then incubated at 4°C overnight with primary antibodies followed by the corresponding secondary antibody. Specific protein bands were detected using avidin-biotinylated horseradish peroxidase in conjunction with an enhanced chemiluminescence detection kit (GE Healthcare, Fairfield, CT, USA). The following antibodies were used in this experiment: *α*-tubulin (Proteintech Group Cat# 11224-1-AP, RRID: AB_2210206, Rosemont, IL, USA), ATGL (Thermo Fisher Scientific Cat# MA5-14990, RRID: AB_10974333, Waltham, MA, USA), cluster of differentiation 36 (CD36) (Abcam Cat# ab133625, RRID: AB_2716564, Cambridge, Cambridgeshire, United Kingdom), glucose transporter-1 (GLUT1) (Abcam Cat# ab115730, RRID: AB_10903230, Cambridge, Cambridgeshire, United Kingdom), glucose transporter-4 (GLUT4) (Santa Cruz Biotechnology Cat# sc-53566, RRID: AB_629533, Santa Cruz, CA, USA), peroxisome proliferator-activated receptor-*α* (PPAR*α*) (Abcam Cat# ab8934, RRID: AB_306869, Cambridge, Cambridgeshire, United Kingdom), and PPAR-*γ* coactivator-1*α* (PGC1*α*) (Santa Cruz Biotechnology Cat# sc-13067, RRID: AB_2166218, Santa Cruz, CA, USA).

### 2.5. Blood Parameters

Blood samples were collected from anesthetized animals by a retroorbital puncture. Serum levels of TG, FFA, aspartate aminotransferase (AST), lactate dehydrogenase (LDH), *α*-hydroxybutyrate dehydrogenase (*α*-HBDH), creatine kinase (CK), creatine kinase-MB (CK-MB), and cardiac troponin T (cTnT) were determined using commercial kits. Fasting blood glucose was monitored using blood glucose strips and the glucometer (Abbott, Chicago, IL, USA).

### 2.6. Tissue Lipid Analysis

Tissue TG and FFA contents were obtained and measured according to the instructions of a TG quantification assay kit (Cat# E1003, Applygen, Beijing, China) and FFA quantification assay kit (Cat# ab65341, Abcam, Cambridge, Cambridgeshire, United Kingdom), respectively.

### 2.7. Echocardiographic Analysis

Mice were anesthetized with a mixture of isoflurane and oxygen. Cardiac function was assessed by echocardiography with a Vivid 7 (GE Medical Systems, Fairfield, CT, USA) instrument. The images were collected from the typical parasternal long axis, apical four-chamber, and apical five-chamber views. GE Medical Systems software was used for data acquisition and subsequent analysis.

### 2.8. Adenosine Triphosphate (ATP) Content

The ATP content in heart tissue was measured according to the instructions of an ATP assay kit (Cat# ab83355, Abcam, Cambridge, Cambridgeshire, United Kingdom).

### 2.9. FAO and Glucose Oxidation

Freshly isolated heart tissues were homogenized in ice-cold buffer (100 mM KCl, 40 mM Tris-HCl, 10 mM Tris base, 5 mM MgCl_2_·6H_2_O, 1 mM EDTA, and 1 mM ATP, pH 7.4) at a 20-fold dilution. Oxidation was measured in a 200 *μ*l reaction mixture containing 100 mM sucrose, 10 mM Tris-HCl, 10 mM KPO_4_, 100 mM KCl, 1 mM MgCl_2_·6H_2_O, 1 mM L-carnitine, 0.1 mM malate, 2 mM ATP, 0.05 mM coenzyme A, and 1 mM dithiothreitol (pH 7.4) with either 1 *μ*Ci [9,10-^3^H(N)] palmitic acid (PerkinElmer, Waltham, MA, USA) and 100 *μ*M unlabeled palmitic acid complexed to BSA or 0.1 *μ*Ci D-[U-^14^C] glucose (PerkinElmer, Waltham, MA, USA) and 200 *μ*M glucose. Oxidation reaction was measured for 30 min at 25°C and stopped by adding 70% perchloric acid. The products were examined by liquid scintillation counting [28].

### 2.10. Gene Expression Analysis

Fresh tissues were removed and immersed in RNA*later™* Solution (Cat# AM7020, Thermo Fisher Scientific, Waltham, MA, USA). Total RNA was extracted with the TRIzol™ reagent (Cat# 15596018, Thermo Fisher Scientific, Waltham, MA, USA). cDNA was prepared from total RNA using a QuantiNova™ reverse transcription kit (Cat# 205411, Qiagen, Hilden, North Rhine-Westphalia, Germany) according to the manufacturer's instructions. Quantitative polymerase chain reaction (PCR) was performed using the QuantiNova™ SYBR® Green PCR kit (Cat# 208054, Qiagen, Hilden, North Rhine-Westphalia, Germany) on the Applied Biosystems® 7500 Real-Time PCR System according to the manufacturer's instructions. The primers were as follows: very long-chain acyl-CoA dehydrogenase (*Acadvl*) forward (5′-CCG GTT CTT TGA GGA AGT GAA-3′) and reverse (5′-AGT GTC GTC CTC CAC CTT CTC-3′), medium-chain acyl-CoA dehydrogenase (*Acadm*) forward (5′-GAT GCA TCA CCC TCG TGT AAC-3′) and reverse (5′-AAG CCC TTT TCC CCT GAA-3′), long-chain acyl-CoA dehydrogenase (*Acadl*) forward (5′-TTT CCG GGA GAG TGT AAG GA-3′) and reverse (5′-ACT TCT CCA GCT TTC TCC CA-3′), acyl-CoA oxidase 1 (*Acox1*) forward (5′-GGG AGT GCT ACG GGT TAC ATG-3′) and reverse (5′-CCG ATA TCC CCA ACA GTG ATG-3′), carnitine palmitoyltransferase 1*α* (*Cpt1α*) forward (5′-TGA GTG GCG TCC TCT TTG G-3′) and reverse (5′-CAG CGA GTA GCG CAT AGT CA-3′), *Cpt1β* forward (5′-GGC ACC TCT TCT GCC TTT AC-3′) and reverse (5′-TTT GGG TCA AAC ATG CAG AT-3′), pyruvate dehydrogenase kinase 4 (*Pdk4*) forward (5′-ATC TAA CAT CGC CAG AAT TAA ACC-3′) and reverse (5′-GGA ACG TAC ACA ATG TGG ATT G-3′), and *18S* forward (5′- CCA TCC AAT CGG TAG TAG CG-3′) and reverse (5′-GTA ACC CGT TGA ACC CCA TT-3′). Relative mRNA levels were quantified and normalized to the levels of 18S rRNA.

### 2.11. Statistical Analysis

Data represent the mean ± standard error of the mean (SEM). Statistical differences between groups were assessed by two-tailed Student's *t-*test or one-way analysis of variance (ANOVA) post hoc tests, as appropriate. For all studies, values of *P* < 0.05 were considered statistically significant. Statistical analyses were performed with the software program IBM SPSS Statistics, version 22 (SPSS Inc.).

## 3. Results

### 3.1. Increased ATGL Expression in the Heart of Burned Mice

**Figure 1:**
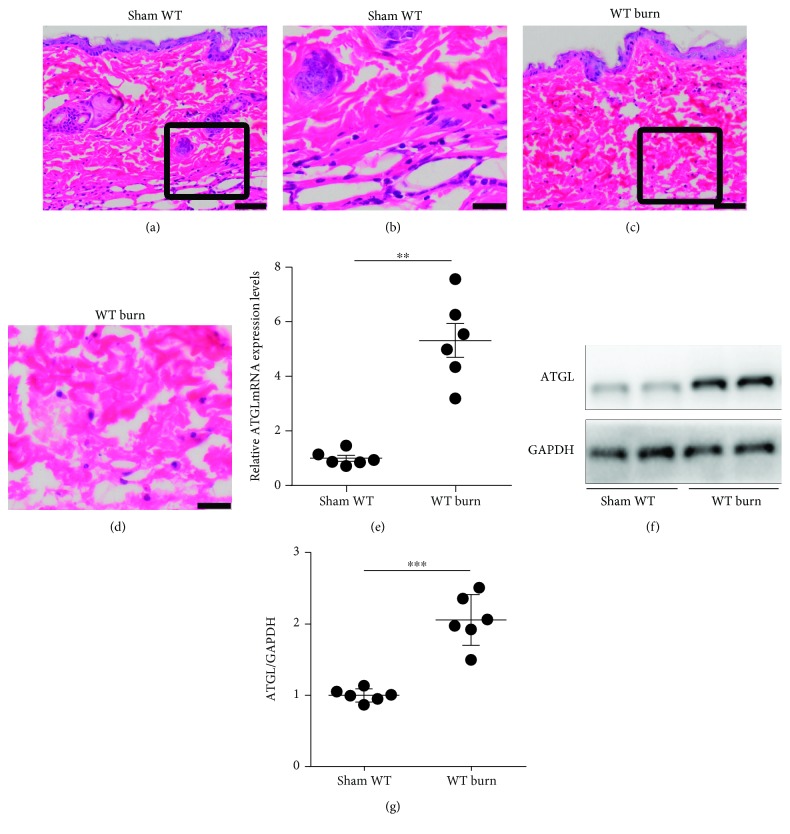
Increased ATGL expression in the heart of burned mice. (a–d) Hematoxylin-eosin staining of skin histological sections with or without burn injury. Panels (b, d) showed high-magnification image of the inset in panels (a, c). Bar: 50 *μ*m (a, c) or 20 *μ*m (b, d). (e) Cardiac ATGL mRNA was detected by reverse transcription-polymerase chain reaction with or without burn injury; *n* = 6. (f, g) Cardiac ATGL protein level was examined by immunoblot analysis; *n* = 6. Data are shown as the mean ± SEM. ^∗∗^ *P* < 0.01 and ^∗∗∗^ *P* < 0.001. *P* values were derived from two-tailed Student's *t*-test.

To discern the change in the cardiac ATGL level after burn challenge, we first constructed a mouse model of third-degree burn injury in approximately 25% of the TBSA. The pathological section showed evidence of a full-thickness skin injury involving epidermis, dermis, and subcutaneous tissue (Figures 1(a)–1(d)). Subsequently, the hearts of burned mice were obtained, and we found that ATGL mRNA and protein expression showed a 5.32-fold and 2.05-fold increase, respectively, compared with that in WT littermates (Figures 1(e)–1(g)).

### 3.2. ATGL Haploinsufficiency Could Not Alleviate Burn-Induced Cardiac Lipid Accumulation

**Figure 2:**
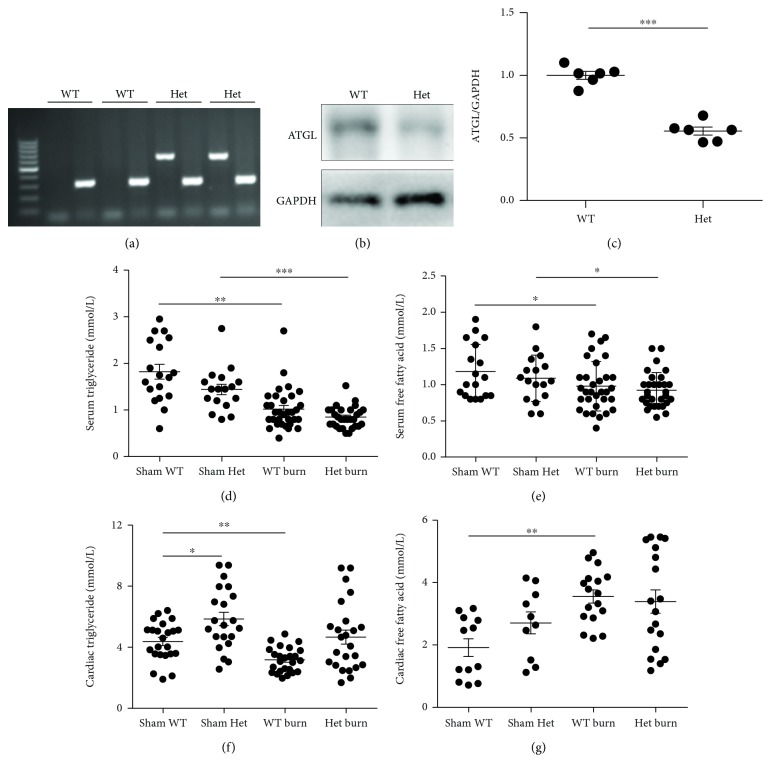
ATGL haploinsufficiency could not alleviate burn-induced cardiac lipid accumulation. (a) Identification of WT and ATGL-Het mice by PCR analysis. (b, c) Immunoblot analysis showing ATGL protein expression in WT and ATGL-Het mice; *n* = 6. (d, e) Analysis comparing serum TG and FFA between WT and ATGL-Het mice with or without burn injury; *n* = 17 − 32. (f, g) Cardiac TG and FFA were compared between WT and ATGL-Het mice with or without burn challenge; *n* = 10 − 25. Het denotes ATGL-Het. Data are shown as the mean ± SEM. ^∗^ *P* < 0.05, ^∗∗^ *P* < 0.01, and ^∗∗∗^ *P* < 0.001. *P* values were derived from two-tailed Student's *t*-test (c) or one-way ANOVA with the appropriate posttest (d–g).

Considering that the excessive FFA accumulation in the heart caused by augmented fat metabolism exhibits multiple toxicities during burn stimulation [11, 12], we first explored whether a reduction in ATGL expression would alleviate the lipid accumulation and have a protective effect on the hearts of burned mice by decreasing TG hydrolysis. ATGL-Het mice (Figure 2(a)) were used instead of ATGL-KO mice to simulate haploinsufficiency of ATGL, as the latter exhibited a lethal effect on the heart at an early age [21]. Significantly decreased ATGL protein expression was shown in the heart of ATGL-Het mice compared with that of WT mice (Figures 2(b) and 2(c)). The fasting blood glucose, serum TG, and FFA levels showed a robust reduction in both WT and ATGL-Het mice after burn challenge, which suggested an increase in glucose, TG, and FFA utilization due to burn injury, but minor changes were detected between WT and ATGL-Het mice under both normal conditions and after burn injury (Figures 2(d) and 2(e) and Figure S1). TG content in the heart tissue of ATGL-Het mice was increased by 1.34-fold compared to that in WT littermates under basal conditions, and the FFA content showed little change. After burn injury, WT mice exhibited a robust decrease in the cardiac TG level, while the cardiac TG level in ATGL-Het mice remained the same, suggesting the suppression of TG hydrolysis in ATGL-Het mice following burn challenge. At the same time, the FFA content in the heart tissue of WT mice showed a marked increase after burn injury, while that in ATGL-Het mice exhibited no statistically significant change, which may be attributed to the inhibition of TG hydrolysis. There was no difference in cardiac TG or FFA content between WT and ATGL-Het mice after burn injury, indicating no alleviation of lipid accumulation with ATGL haploinsufficiency (Figures 2(f) and 2(g)). Thus, partial inhibition of ATGL deficiency-mediated TG hydrolysis could not improve cardiac lipid accumulation after burn injury; however, whether the strategy could protect against burn-induced cardiac dysfunction needs to be further explored.

### 3.3. Partial Loss of ATGL Does Not Protect against Burn-Induced Cardiac Dysfunction

In vivo cardiac function was evaluated by echocardiographic analysis (Table 1). In the absence of burn injury, WT and ATGL-Het mice showed no significant differences in cardiac systolic and diastolic functions. After burn challenge, the WT mice exhibited a robust decrease in ejection fraction (EF), fractional shortening (FS), and velocity of circumference (VCF), indicating cardiac systolic dysfunction. Cardiac diastolic function also worsened after burn injury, as shown by a decreased peak *E* wave at the septal annulus at early diastole relative to the peak *A* wave at the septal annulus at late diastole, an increased deceleration time of mitral inflow *E* (EDT), and an increased isovolumetric relaxation time (IVRT). The EF, FS, and VCF, which reflect cardiac systolic function, displayed minor improvements in ATGL-Het mice compared with WT littermates after burn challenge. Diastolic function worsened in ATGL-Het mice compared with WT mice after burn stimulation, as shown by increased EDT and IVRT (Table 1). Hence, partial loss of ATGL does not protect against burn-induced cardiac dysfunction.

**Figure 3:**
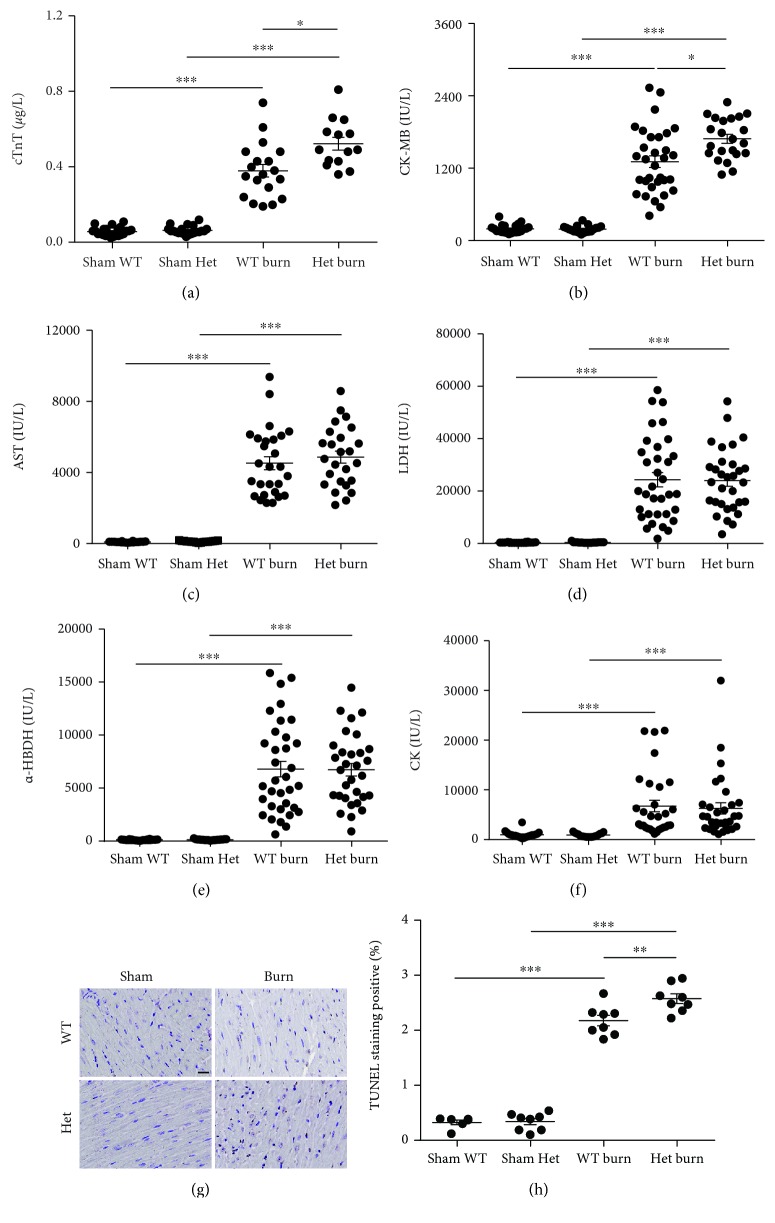
Partial loss of ATGL does not protect against burn-induced cardiac dysfunction. (a–f) Blood markers of cardiac damage were compared between WT and ATGL-Het mice with or without burn challenge; *n* = 14 − 34. (g, h) Representative images and quantitative analysis showing cardiac apoptosis using TUNEL staining in WT and ATGL-Het mice with or without burn injury. Bar: 20 *μ*m; *n* = 8. Data are shown as the mean ± SEM. ^∗^ *P* < 0.05, ^∗∗^ *P* < 0.01, and ^∗∗∗^ *P* < 0.001. *P* values were derived from one-way ANOVA with the appropriate posttest.

In addition, the serum markers of myocardial injury, including cTnT, AST, *α*-HBDH, CK-MB, CK, and LDH, were not significantly different between ATGL-Het mice and WT littermates in the absence of burn injury. However, following burn injury, the levels of serum CK-MB and cTnT were strongly increased in ATGL-Het mice compared to WT littermates (Figures 3(a) and 3(b)), while the levels of other injury markers, including AST, *α*-HBDH, CK, and LDH, were comparable between ATGL-Het mice and WT mice (Figures 3(c)–3(f)). Moreover, cardiomyocyte apoptosis was shown to be elevated in ATGL-Het mice compared to WT littermates after burn challenge (Figures 3(g) and 3(h)). Taken together, these findings demonstrate that partial reduction of cardiac ATGL expression did not protect against, but rather aggravated, burn-induced myocardial impairment.

### 3.4. Cardiac-Specific ATGL Overexpression Prevents Lipid Accumulation following Burn Injury

**Figure 4:**
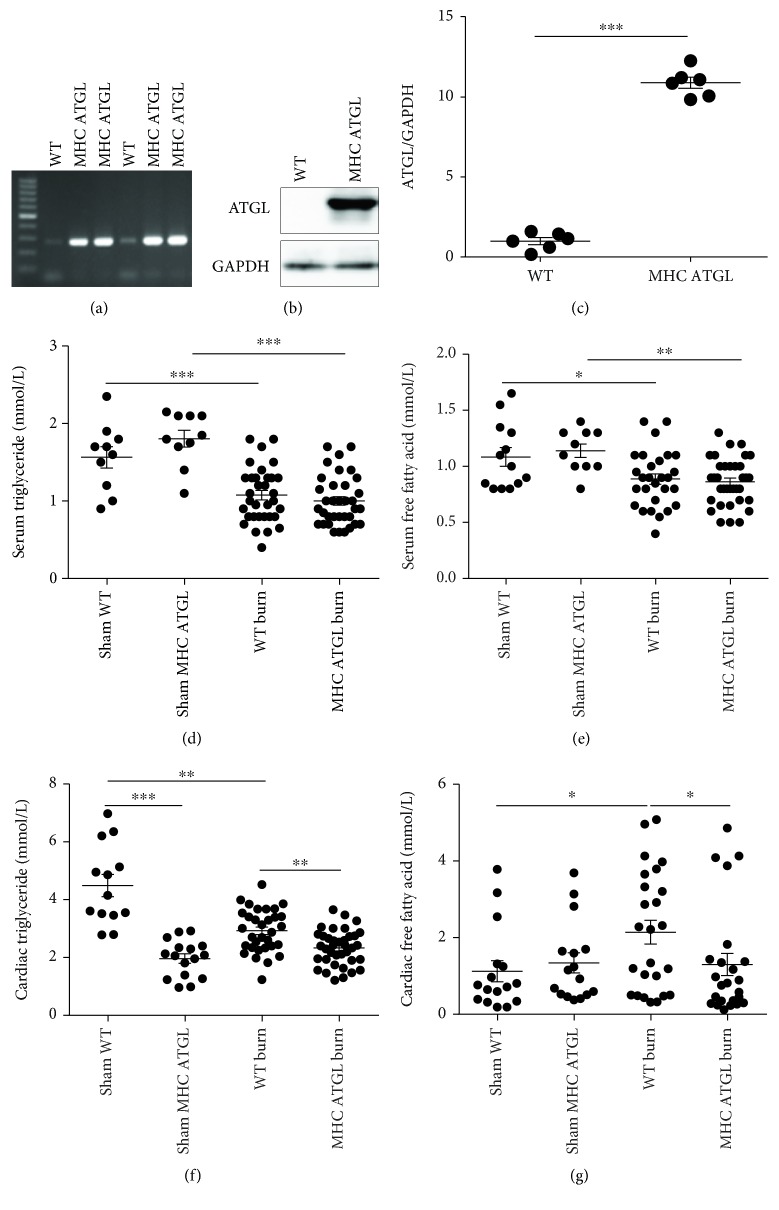
Cardiac-specific ATGL overexpression prevents lipid accumulation following burn injury. (a) Identification of WT and MHC-ATGL Het mice by PCR analysis. (b, c) Immunoblot analysis showing ATGL expression in WT and MHC-ATGL Het mice under basal conditions; *n* = 6. (d, e) Analysis comparing serum TG and FFA between WT and MHC-ATGL Het mice with or without burn injury; *n* = 10 − 35. (f, g) Cardiac TG and FFA were compared between WT and MHC-ATGL Het mice with or without burn stimulation; *n* = 13 − 37. MHC-ATGL denotes MHC-ATGL Het. Data are shown as the mean ± SEM. ^∗^ *P* < 0.05, ^∗∗^ *P* < 0.01, and ^∗∗∗^ *P* < 0.001. *P* values were derived from two-tailed Student's *t*-test (c) and one-way ANOVA with the appropriate posttest (d–g).

Based on the results showing that partial loss of ATGL did not alleviate burn-induced lipid accumulation, cardiomyocyte apoptosis, and cardiac dysfunction, we investigated whether ATGL overexpression would exert protective effects. To test the hypothesis, MHC-ATGL Het mice were used to clarify the effect of elevated ATGL expression on cardiac metabolism and function with or without burn injury (Figures 4(a)–4(c)). The results showed that fasting blood glucose, serum TG, and FFA levels were decreased after burn injury in both WT and MHC-ATGL Het mice and that there were minor differences between those mice with or without burn injury (Figures 4(d) and 4(e) and Figure S1). In contrast to ATGL-Het mice, MHC-ATGL Het mice displayed a reduction in cardiac TG either with or without burn challenge compared to WT littermates (Figure 4(f)). In addition, MHC-ATGL Het mice showed a level of FFA comparable to that in WT littermates prior to burn injury, but the level of FFA was significantly decreased in MHC-ATGL Het mice compared to WT littermates after burn injury (Figure 4(g)). Altogether, these data show that cardiac ATGL overexpression could protect against cardiac lipid accumulation after burn injury.

### 3.5. MHC-ATGL Mice Are Resistant to Burn-Induced Cardiac Injury

We then investigated whether MHC-ATGL Het mice exhibited improved cardiac function and cardiomyocyte survival compared with WT littermates following burn injury. Both systolic and diastolic functions were comparable between WT and MHC-ATGL Het mice under basal conditions (Table 2). However, in the presence of burn injury, a significant improvement of both systolic and diastolic functions was detected in MHC-ATGL Het mice compared with WT littermates (Table 2).

**Figure 5:**
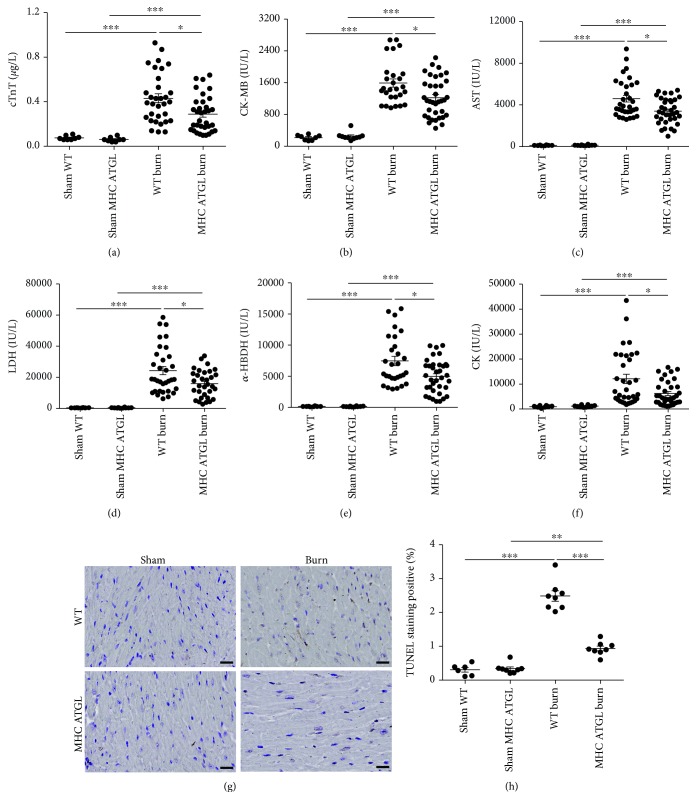
MHC-ATGL mice are resistant to burn-induced cardiac injury. (a–f) Blood markers of cardiac injury were compared between WT and MHC-ATGL Het mice with or without burn challenge; *n* = 8 − 36. (g, h) Representative images and quantitative analysis showing cardiac apoptosis using TUNEL staining in WT and MHC-ATGL Het mice with or without burn challenge. Bar: 20 *μ*m; *n* = 8. Data are shown as the mean ± SEM. ^∗^ *P* < 0.05, ^∗∗^ *P* < 0.01, and ^∗∗∗^ *P* < 0.001. *P* values were derived from one-way ANOVA with the appropriate posttest.

The concentrations of serum AST, *α*-HBDH, LDH, CK, CK-MB, and cTnT (Figures 5(a)–5(f)), as well as the cardiomyocyte apoptosis rate (Figures 5(g) and 5(h)), showed minor differences between MHC-ATGL Het mice and WT littermates in the absence of burn injury but were significantly reduced in MHC-ATGL Het mice compared to WT mice after burn challenge, suggesting the protective effect of cardiac ATGL overexpression on burn-induced myocardial injury. Thus, these findings indicated that cardiac-specific overexpression of ATGL protected mice from burn-induced cardiac injury and improved cardiac function.

### 3.6. Role of ATGL in Fat and Glucose Metabolisms

**Figure 6:**
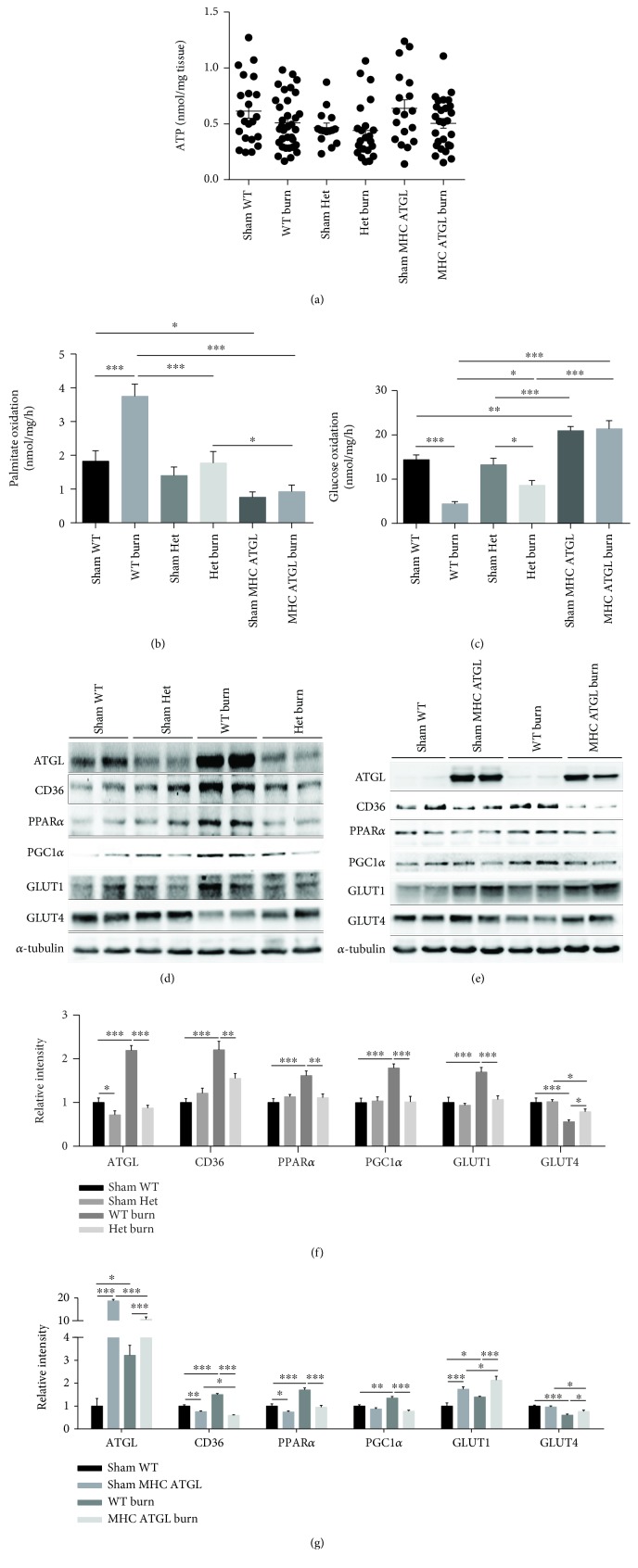
Role of ATGL in fat and glucose metabolisms. (a) ATP content of the heart was detected in ATGL-Het, MHC-ATGL Het, and WT mice before or after burn challenge; *n* = 15 − 34. (b, c) Palmitate oxidation and glucose oxidation were compared between the different genotypes of mice with or without burn challenge; *n* = 8. (d–g) Protein expression of ATGL, CD36, PPAR*α*, PGC1*α*, GLUT1, and GLUT4 in WT, ATGL-Het, and MHC-ATGL Het mice was detected by immunoblot analysis with or without burn challenge; *n* = 6. Data are shown as the mean ± SEM. ^∗^ *P* < 0.05, ^∗∗^ *P* < 0.01, and ^∗∗∗^ *P* < 0.001. *P* values were derived from one-way ANOVA with the appropriate posttest.

The above results showed that MHC-ATGL Het mice were more resistant to burn-induced cardiac lipid accumulation, apoptosis, and dysfunction than WT littermates, while partial loss of ATGL aggravated the burn-induced injuries compared with those in the WT littermates. As ATGL is an essential metabolic enzyme, alterations in ATGL-mediated cardiac metabolism might underlie the different outcomes of the two mouse models following burn challenge. To elucidate the discrepant metabolic pattern of the two mouse models, we first examined the ATP content in heart tissue. We found that the ATP content was comparable among ATGL-Het, MHC-ATGL Het, and WT mice before and after burn challenge (Figure 6(a)). Both MHC-ATGL Het and ATGL-Het mice displayed reduced FAO compared with WT mice after burn challenge, and the FAO level in MHC-ATGL Het mice was even lower than that in ATGL-Het mice (Figure 6(b)). On the other hand, MHC-ATGL Het mice exhibited more elevated glucose oxidation levels than WT littermates or ATGL-Het mice in the absence or presence of burn challenge, indicating a switch from FAO to glucose oxidation for energy in MHC-ATGL Het mice with or without burn challenge (Figure 6(c)). In contrast, the glucose oxidation level in ATGL-Het mice, which was comparable to that in WT littermates prior to burn injury, decreased significantly after burn challenge but was still higher than that in burned WT mice (Figure 6(c)). These data indicated a crucial role of ATGL in the modulation of fat and glucose metabolisms: FAO was inhibited in both MHC-ATGL Het and ATGL-Het mice after burn challenge, and ATGL overexpression increased glucose oxidation levels under normal conditions and after burn injury. After burn injury, glucose oxidation was less suppressed in ATGL-Het mice than in WT littermates but could not be sufficiently activated to compensate for the decrease in FAO (Figures 6(b) and 6(c)).

However, the mechanism of how ATGL mediated the changes in fat and glucose metabolisms remained unclear. To further discern the metabolic processes in detail, we detected the markers of biochemical pathways that regulate glucose and fat utilization [29–31]. Regarding markers of fat metabolism, the expression of CD36, PPAR*α*, and PGC1*α* increased in the heart of WT mice after burn challenge, which may account for the increase in fatty acid (FA) uptake and FAO after burn injury. MHC-ATGL Het mice exhibited a decrease in CD36 and PPAR*α* expressions at the baseline, and CD36 expression further decreased after burn injury. ATGL-Het mice exhibited protein levels of CD36, PPAR*α*, and PGC1*α* that were comparable to those in WT mice, but their levels were much lower after burn injury in ATGL-Het mice than in WT mice (Figures 6(d)–6(g)). These data indicated that FA uptake and FAO were reduced in MHC-ATGL Het mice compared with WT littermates under normal conditions and that after burn injury, fat metabolism was further inhibited in both ATGL-Het and MHC-ATGL Het mice.

Regarding markers of glucose utilization, the expression of GLUT1 increased while that of GLUT4 decreased sharply in WT mice after burn challenge, which may account for the reduction in glucose oxidation after burn injury (Figure 6(c)). An increase in the GLUT1 protein level was detected in MHC-ATGL Het mice compared with WT littermates; the GLUT1 protein level was further elevated after burn injury and remained higher in burned MHC-ATGL Het mice than in burned WT mice. GLUT4 protein expression in MHC-ATGL Het mice decreased after burn injury but remained higher than that in burned WT mice. These data suggested that the increase in glucose utilization in the hearts of MHC-ATGL Het mice may depend on the elevation in GLUT1 expression and the relatively reduced inhibition of GLUT4 expression. ATGL-Het mice exhibited comparable protein levels of GLUT1 and GLUT4 at the baseline. The increase in GLUT1 expression induced by burn injury was partly inhibited by ATGL deletion, and GLUT4 expression was also decreased after burn injury, although it remained higher than that in burned WT mice (Figures 6(d)–6(g)).

### 3.7. ATGL Regulates the Expression of Genes Involved in FA and Glucose Metabolisms

**Figure 7:**
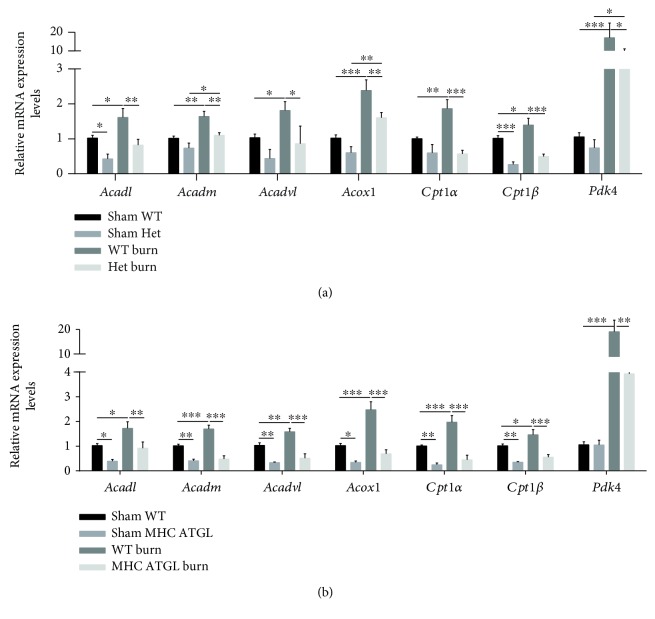
ATGL regulates the expression of genes involved in FA and glucose metabolisms. (a) Genetic testing was performed in WT and ATGL-Het mice with or without burn injury. (b) Genetic testing was performed in WT and MHC-ATGL Het mice with or without burn stimulation. Relative mRNA levels were quantified and normalized to the levels of *18S*; *n* = 5 − 9. Data are shown as the mean ± SEM. ^∗^ *P* < 0.05, ^∗∗^ *P* < 0.01, and ^∗∗∗^ *P* < 0.001. *P* values were derived from one-way ANOVA with the appropriate posttest.

Genetic testing was further applied to identify changes in fat and glucose metabolisms. Among the tested genes, *Acadl*, *Acadm*, *Acadvl*, *Acox1*, *Cpt1α*, and *Cpt1β* are involved in mitochondrial FA *β*-oxidation. *Pdk4* inhibits pyruvate dehydrogenase activity, which downregulates aerobic respiration and inhibits the formation of acetyl-coenzyme A from pyruvate, thereby decreasing glucose utilization and increasing fat metabolism. Burn challenge promoted the expressions of *Acadl*, *Acadm*, *Acadvl*, *Acox1*, *Cpt1α*, and *Cpt1β*, and *Pdk4* expression levels were also elevated, suggesting increased fat metabolism and decreased glucose utilization compared to those in WT littermates at the baseline (Figures 7(a) and 7(b)), which was consistent with the results in vitro (Figures 6(b) and 6(c)). In MHC-ATGL Het mice, *Acadl*, *Acadm*, *Acadvl*, *Acox1*, *Cpt1α*, and *Cpt1β* expression levels were all decreased compared to those in WT littermates, and the *Pdk4* expression level was similar to those in WT littermates; burn challenge did not further promote their expression. The expression levels of all the above genes were significantly decreased following burn injury in MHC-ATGL Het mice compared with WT littermates, indicating suppressed FA *β*-oxidation and increased glucose utilization induced by ATGL overexpression (Figure 7(b)). In ATGL-Het mice, the expression of *Cpt1β* and *Acadl* was decreased, while the expression of other genes was comparable to that in WT littermates. After burn injury, *Acadm*, *Acox1*, and *Pdk4* levels increased in ATGL-Het mice, but the expression level of all those genes was robustly decreased compared with that in burned WT littermates, indicating reduced FA *β*-oxidation with a compensatory increase in glucose utilization (Figure 7(a)).

## 4. Discussion

Serious burn challenge induces cardiomyopathy development, which is a major risk factor for the development of sepsis, multiple organ failure, and death; however, the potential mechanisms remain elusive. Maladaptive cardiac metabolism is an important trigger of cardiac dysfunction. The salient findings from our present study were that cardiac-specific overexpression of ATGL, the key enzyme that hydrolyzes TG at the initial step in fat catabolic metabolism, initiated a critical protective effect against cardiac lipid accumulation, cardiomyocyte apoptosis, and cardiac dysfunction after burn challenge, while partial loss of ATGL aggravated such injuries and cardiac dysfunction.

The beating heart has a very high energy demand and has thus evolved to utilize a variety of energy sources for ATP production [31]. The healthy adult heart derives most of its energy from FAO [32]. While FAO produces more ATP, it comes at the cost of increased oxygen consumption, thereby making FAO less efficient than glucose oxidation from a true energetics stand point [33]. In addition, FAs can induce mitochondrial uncoupling and lipid accumulation, leading to less efficient ATP production [34, 35]. Following burn injury, more TG is hydrolyzed to produce sufficient FA substrates for FAO [36, 37]. Given the increased risk of cardiac injury and dysfunction in patients with serious burns, it is indeed tempting to hypothesize that high FAO has deleterious effects on the heart. Whether inhibition of TG hydrolysis and FAO and/or an increase in glucose utilization would be beneficial for the heart and how to induce changes in metabolic pathways need to be further studied; the results of these future studies may contribute to the prevention and treatment of cardiac injury postburn.

In the current study, we addressed for the first time the changes in cardiac ATGL expression after burn challenge and explored whether regulation of ATGL expression could alleviate the burn-induced cardiac injury by modulating fat and glucose metabolisms. We found that burn challenge augmented FA metabolism but not glucose metabolism, which was accompanied by significantly elevated cardiac ATGL mRNA and protein expression. The ATGL-Het mice, which were used to mimic partial loss of cardiac ATGL, showed unrelieved cardiac lipid accumulation, cardiomyocyte apoptosis, and cardiac dysfunction after burn injury, which may be due to the suppression of cardiac FA transport and FAO, and the relatively restrained glucose utilization following burn challenge. On the other hand, overexpression of ATGL ameliorated burn-induced cardiomyopathy, as indicated by decreased cardiomyocyte apoptosis and serum myocardial enzyme level, and improved in vivo cardiac function, which may be mediated by the shift in substrate preference away from FAs towards enhanced glucose utilization. The shift in metabolic processes may have a protective effect via a reduction in oxygen consumption and the accumulation of harmful catabolites [38].

The cardioprotective effect of ATGL overexpression on burn injury was hypothesized to be attributed to the metabolic shift towards glucose utilization and the reduction in cardiac lipid accumulation, but the underlying mechanism remains unknown. An increase in glucose uptake mediated by GLUT1 and GLUT4 upregulation and an increase in glucose oxidation, as indicated by *Pdk4* downregulation, were noted in the burned MHC-ATGL Het mice, along with reduced FA transportation, as shown by decreased CD36, *Cpt1α*, and *Cpt1β* expression, and FAO, as indicated by downregulation of PPAR*α*/PGC1*α*, *Acad*, and *Acox1* expression, compared to those in burned WT mice. It should be noted that MHC-ATGL Het mice exhibited a very low level of FA uptake in the absence of burn injury, as manifested by reduced expression of CD36, which led to decreased intracellular FFA overload although the level of FAO was decreased. The combined alterations of these metabolic genes may explain the shift in cardiac substrate metabolism and alleviated lipid accumulation induced by ATGL overexpression.

On the other hand, although ATGL-Het mice displayed levels of FAO and glucose oxidation comparable to those in WT mice under normal conditions, FAO or glucose oxidation was suppressed following burn challenge in ATGL-Het mice compared to WT and MHC-ATGL Het mice, indicating maladaptive and adverse metabolic changes. The downregulation of CD36, *Cpt1α*, and *Cpt1β*, which indicated inhibited FA transport, and the decrease in PPAR*α*/PGC1*α*, *Acad*, and *Acox1* expression, which suggested inhibited FAO, underlie the decrease in fat metabolism in ATGL-Het mice compared with WT mice after burn challenge, which was consistent with the tendency observed in MHC-ATGL Het mice after burn challenge. However, unlike MHC-ATGL Het mice, ATGL-Het mice exhibited CD36 expression levels comparable to those in WT littermates under basal conditions, indicating a relatively high level of FA uptake in ATGL-Het mice compared with MHC-ATGL Het mice, which may account for the increased FFA level in the heart tissue of ATGL-Het mice. Furthermore, GLUT1 expression was significantly inhibited in the ATGL-Het mice compared to the WT mice postburn, and although GLUT4 expression was increased to compensate for the inhibited fat metabolism, it was still lower than that prior to burn injury, indicating that GLUT1 expression was inhibited while GLUT4 expression was not sufficiently activated by the burn challenge. Thus, the changes in differentially expressed proteins may explain the suppression of fat metabolism and the deficiency of the compensatory increase in glucose utilization, which led to aggravated burn-induced cardiac injury in ATGL-Het mice.

In our study, it was an unexpected outcome that as an important lipase, ATGL overexpression decreased FAO and elevated glucose oxidation with or without burn injury. Although a similar result was also reported in previous study [39], the mechanism was far from clear. An important finding of this study was that the increased GLUT1 expression level in MHC-ATGL Het mice, which was consistent with previous study [39], was the critical factor that led to a shift in cardiac substrate preference towards a greater reliance on glucose and to the concomitant suppression of FAO. This metabolic shift has long been interpreted as an oxygen-sparing mechanism of the heart. Since FA metabolism was inhibited in both MHC-ATGL Het and ATGL-Het mice following burn injury, whereas GLUT1 expression was increased in MHC-ATGL Het mice, we inferred that GLUT1 elevation was the downstream effector of ATGL overexpression and not a compensatory response to suppressed fat metabolism. We have tried to test the hypothesis at the cellular level. As the survival time of primary cultured cardiomyocytes is limited, H9C2 cells transfected with ATGL lentivirus were used as a cell model of stable ATGL overexpression, and GLUT1 expression was detected. Unfortunately, GLUT1 expression did not increase as expected in the H9C2 cells transfected with ATGL lentivirus (data not shown). The cell type applied (H9C2 cells could not properly simulate primary cardiomyocytes), lentivirus transfection time, and differences between the in vitro and in vivo conditions should be taken into account when considering the negative results. Although we have not clarified why myocardial GLUT1 expression was upregulated in response to ATGL overexpression, these data have solidly demonstrated that ATGL is not only an important enzyme in fat metabolism but also a key regulator of glucose utilization.

## 5. Conclusions

Here in the present study, we demonstrated for the first time that elevated cardiac ATGL expression following burn injury is an adaptive, albeit insufficient, response to compensate for the increase in energy consumption, and further augmentation of ATGL expression would help ameliorate burn-induced cardiac injury.

## Figures and Tables

**Table 1:** In vivo cardiac function of WT and ATGL-Het mice with or without burn injury.

	Sham		Burn	
	WT	ATGL-Het	WT	ATGL-Het
HR (bpm)	385 ± 9	362 ± 5	364 ± 8	364 ± 5
IVSs (mm)	1.19 ± 0.02	1.16 ± 0.04	1.19 ± 0.04	1.11 ± 0.03
IVSd (mm)	0.87 ± 0.06	0.97 ± 0.07	0.93 ± 0.04	0.83 ± 0.03
LVPWs (mm)	1.20 ± 0.04	1.18 ± 0.05	1.26 ± 0.03	1.12 ± 0.05
LVPWd (mm)	0.98 ± 0.09	0.93 ± 0.05	0.91 ± 0.03	0.85 ± 0.03
LVIDs (mm)	1.34 ± 0.08	1.30 ± 0.07	1.88 ± 0.05^a^	1.89 ± 0.08^b^
LVIDd (mm)	3.03 ± 0.10	2.82 ± 0.06	3.01 ± 0.08	2.91 ± 0.07
EF (%)	91.13 ± 1.01	89.13 ± 1.26	76.22 ± 1.10^a^	75.42 ± 0.97^b^
FS (%)	56.00 ± 1.63	52.63 ± 2.10	38.44 ± 0.96^a^	37.37 ± 0.84^b^
VCF (cir/s)	7.49 ± 0.38	7.21 ± 0.22	4.80 ± 0.24^a^	4.98 ± 0.30^b^
*E* velocity (m/s)	0.74 ± 0.03	0.68 ± 0.03	0.60 ± 0.02^a^	0.59 ± 0.03^b^
*A* velocity (m/s)	0.58 ± 0.04	0.53 ± 0.03	0.48 ± 0.02^a^	0.49 ± 0.03
EDT (ms)	9.33 ± 0.67	10.71 ± 0.52	11.56 ± 0.39^a^	14.50 ± 0.60^c^
*E*/*A*	1.31 ± 0.07	1.25 ± 0.03	1.24 ± 0.02	1.22 ± 0.02
*E*_sa_/*A*_sa_	1.43 ± 0.06	1.39 ± 0.03	0.58 ± 0.04^a^	0.55 ± 0.04^b^
IVRT (ms)	15.06 ± 0.71	17.94 ± 1.00	18.83 ± 1.60^a^	22.37 ± 1.37^c^
IVCT (ms)	13.50 ± 2.51	14.94 ± 0.55	13.44 ± 1.00	16.85 ± 0.87
Tei index	0.39 ± 0.04	0.46 ± 0.03	0.42 ± 0.03	0.47 ± 0.05
ET (ms)	75.50 ± 3.31	73.09 ± 2.32	78.78 ± 2.81	72.25 ± 2.93

*A* velocity: mitral *A* velocity; *E* velocity: mitral *E* velocity; *E*/*A*: mitral *E* velocity relative to mitral *A* velocity; *E*_sa_/*A*_sa_: peak *E* wave of early diastolic septal annulus relative to peak *A* wave of late diastolic septal annulus; ET: ejection time; HR: heart rate; IVSs: interventricular septal diameter at systole; IVSd: interventricular septal diameter at diastole; IVCT: intraventricular contraction time; LVPWs: left ventricular posterior wall thickness at systole; LVPWd: left ventricular posterior wall thickness at diastole; LVIDs: left ventricular internal diameter at systole; LVIDd: left ventricular internal diameter at diastole. Data are shown as the mean ± SEM (^a^ *P* < 0.05 vs. sham WT, ^b^ *P* < 0.05 vs. sham ATGL-Het, and ^c^ *P* < 0.05 vs. all groups). *P* values were derived from one-way ANOVA with the appropriate posttests.

**Table 2:** In vivo cardiac function of WT and MHC-ATGL Het mice with or without burn injury.

	Sham		Burn	
	WT	MHC-ATGL Het	WT	MHC-ATGL Het
HR (bpm)	378 ± 6	366 ± 5	366 ± 7	362 ± 6
IVSs (mm)	1.19 ± 0.01	1.12 ± 0.04	1.19 ± 0.04	1.15 ± 0.07
IVSd (mm)	0.85 ± 0.06	0.87 ± 0.03	0.92 ± 0.04	0.84 ± 0.03
LVPWs (mm)	1.21 ± 0.03	1.31 ± 0.03	1.25 ± 0.03	1.33 ± 0.09
LVPWd (mm)	0.93 ± 0.09	0.77 ± 0.03	0.92 ± 0.03	0.87 ± 0.05
LVIDs (mm)	1.32 ± 0.06	1.15 ± 0.10	1.91 ± 0.05^a^	1.71 ± 0.08^b^
LVIDd (mm)	3.02 ± 0.09	2.79 ± 0.05	3.03 ± 0.07	3.01 ± 0.09^b^
EF (%)	91.26 ± 0.87	92.19 ± 1.61	75.60 ± 1.17^c^	84.06 ± 1.12^c^
FS (%)	56.11 ± 1.44	59.30 ± 3.17	37.90 ± 1.02^c^	46.45 ± 1.47^c^
VCF (cir/s)	7.60 ± 0.31	7.97 ± 0.47	4.83 ± 0.21^c^	5.85 ± 0.28^c^
*E* velocity (m/s)	0.73 ± 0.02	0.70 ± 0.02	0.59 ± 0.02^c^	0.66 ± 0.03^d^
*A* velocity (m/s)	0.58 ± 0.03	0.58 ± 0.03	0.49 ± 0.02^a^	0.53 ± 0.03
EDT (ms)	9.00 ± 0.65	7.61 ± 0.44	11.57 ± 0.35^c^	9.47 ± 0.59^d^
*E*/*A*	1.29 ± 0.06	1.22 ± 0.02	1.22 ± 0.03	1.25 ± 0.02
*E*_sa_/*A*_sa_	1.43 ± 0.05	1.32 ± 0.04	0.57 ± 0.03^c^	0.73 ± 0.05^c^
IVRT (ms)	15.61 ± 0.83	14.70 ± 0.86	19.15 ± 1.46^c^	15.50 ± 0.54^d^
IVCT (ms)	13.89 ± 2.25	12.80 ± 1.15	13.75 ± 0.95	13.80 ± 1.44
Tei index	0.40 ± 0.04	0.46 ± 0.05	0.44 ± 0.03	0.39 ± 0.03
ET (ms)	73.89 ± 2.44	74.67 ± 1.97	77.40 ± 2.87	74.73 ± 2.19

Data are shown as the mean ± SEM (^a^ *P* < 0.05 vs. sham WT, ^b^ *P* < 0.05 vs. sham MHC-ATGL Het, ^c^ *P* < 0.05 vs. all groups, and ^d^ *P* < 0.05 vs. WT burn). *P* values were derived from one-way ANOVA with the appropriate posttests.

## Data Availability

The datasets used or analyzed during the current study are available from the corresponding authors on reasonable request. All data generated or analyzed during this study are included in the present article.

## Supplementary Materials

Supplementary Materials Figure S1: detection of fasting blood glucose.
