# Supplementary material for: Myocardial Adipose Triglyceride Lipase Overexpression Protects against Burn-Induced Cardiac Lipid Accumulation and Injury
Source: Oxid Med Cell Longev. 2019 May 16;2019:6428924. doi: 10.1155/2019/6428924 (PMC6541965; doi:10.1155/2019/6428924)
Supplement: Supplementary Materials — Figure S1: detection of fasting blood glucose. [file 6428924.f1.pdf]

Myocardial adipose triglyceride lipase overexpression protects against burn-induced cardiac lipid accumulation and injury

Lingfei Li[1,2], Xingyue Zhang[1,2], Qiong Zhang[1,2], Jiezhi Jia[1,2], Junhui Zhang[1,2], Dongxia Zhang[1,2], Huapei Song[1,2], Bing Chen[3], Jiongyu Hu[2,3*], Yuesheng Huang[1,2*]

[1]Institute of Burn Research, Southwest Hospital, Third Military Medical University (Army Medical University), Chongqing, China.
[2]State Key Laboratory of Trauma, Burns and Combined Injury, Southwest Hospital, Third Military Medical University (Army Medical University), Chongqing, China.
[3]Endocrinology Department, Southwest Hospital, Third Military Medical University (Army Medical University), Chongqing, China.

*Corresponding authors:
Yuesheng Huang, Institute of Burn Research, Southwest Hospital, Third Military Medical University (Army Medical University), Chongqing, 400038, China. Phone and fax: +86 023 68766023. E-mail: yshuang1958@163.com;
Jiongyu Hu, Endocrinology Department, Southwest Hospital, Third Military Medical University (Army Medical University), Chongqing, 400038, China. Phone and fax: +86 023 68773162. E-mail: jiongyuhu@163.com.

Supplementary Information

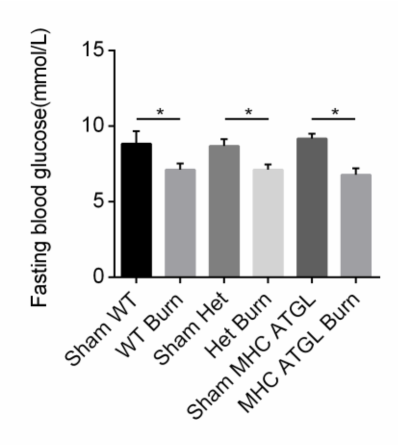

Supplementary Figure S1 Detection of fasting blood glucose. Fasting blood glucose (fasting 8 hours before measurement) was determined between WT, ATGL-Het and MHC-ATGL mice with or without burn injury. n = 8. Data are shown as the mean ± SEM. *$P < 0.05$. *P* values were derived from one-way ANOVA with the appropriate posttest.
